# Protection against SARS-CoV-2 infection by a mucosal vaccine in rhesus macaques

**DOI:** 10.1172/jci.insight.148494

**Published:** 2021-04-28

**Authors:** Yongjun Sui, Jianping Li, Roushu Zhang, Sunaina Kiran Prabhu, Hanne Andersen, David Venzon, Anthony Cook, Renita Brown, Elyse Teow, Jason Velasco, Jack Greenhouse, Tammy Putman-Taylor, Tracey-Ann Campbell, Laurent Pessaint, Ian N. Moore, Laurel Lagenaur, Jim Talton, Matthew W. Breed, Josh Kramer, Kevin W. Bock, Mahnaz Minai, Bianca M. Nagata, Mark G. Lewis, Lai-Xi Wang, Jay A. Berzofsky

**Affiliations:** 1 Vaccine Branch, Center for Cancer Research, National Cancer Institute, NIH, Bethesda, Maryland, USA.; 2 Department of Chemistry and Biochemistry, University of Maryland, College Park, Maryland, USA.; 3 BIOQUAL Inc., Rockville, Maryland, USA.; 4 Biostatistics and Data Management Section, Center for Cancer Research, National Cancer Institute, NIH, Bethesda, Maryland, USA.; 5 Infectious Disease Pathogenesis Section, National Institute of Allergy and Infectious Diseases, NIH, Rockville, Maryland, USA.; 6 Alchem Laboratories Corporation, Alachua, Florida, USA.; 7 Laboratory Animal Sciences Program, Frederick National Laboratory for Cancer Research, Bethesda, Maryland, USA.

**Keywords:** COVID-19, Vaccines, Adaptive immunity, Innate immunity

## Abstract

Effective SARS-CoV-2 vaccines are urgently needed. Although most vaccine strategies have focused on systemic immunization, here we compared the protective efficacy of 2 adjuvanted subunit vaccines with spike protein S1: an intramuscularly primed/boosted vaccine and an intramuscularly primed/intranasally boosted mucosal vaccine in rhesus macaques. The intramuscular-alum–only vaccine induced robust binding and neutralizing antibody and persistent cellular immunity systemically and mucosally, whereas intranasal boosting with nanoparticles, including IL-15 and TLR agonists, elicited weaker T cell and Ab responses but higher dimeric IgA and IFN-α. Nevertheless, following SARS-CoV-2 challenge, neither group showed detectable subgenomic RNA in upper or lower respiratory tracts versus naive controls, indicating full protection against viral replication. Although mucosal and systemic protective mechanisms may differ, results demonstrate both vaccines can protect against respiratory SARS-CoV-2 exposure. In summary, we have demonstrated that the mucosal vaccine was safe after multiple doses and cleared the input virus more efficiently in the nasal cavity and thus may act as a potent complementary reinforcing boost for conventional systemic vaccines to provide overall better protection.

## Introduction

Severe acute respiratory syndrome coronavirus-2 (SARS-CoV-2), the virus responsible for the coronavirus disease 2019 (COVID-19) pandemic, has caused an unprecedented public health crisis. The current pandemic highlighted the need for effective vaccines to reduce the spread of virus. Multiple vaccine strategies, including adenovirus-vectored, inactivated virus, DNA- and mRNA-based platforms, and recombinant viral subunits/protein, are under study to develop safe and effective vaccines against viral transmission and COVID-19 disease (1–8).

Most strategies in clinical trials are focused on systemically administered vaccines, and their ability to induce respiratory mucosal immunity is unknown. Mucosal immunity is important for COVID-19 because the virus infects via the angiotensin-converting enzyme 2 receptor primarily through the upper and lower respiratory tracts (9–11). Recently, the need for mucosal immunity and mucosal vaccines for SARS-CoV-2 has been emphasized (12). Several studies have used intranasal (IN) vaccination to address this option in the current COVID-19 outbreak (13–16). Most of these studies have used adenovirus- or lentiviral vector-based vaccines in rodents and ferrets. Feng and colleagues described the effect of adenovirus 5-spike vaccination following a single IN vaccination followed by SARS-CoV-2 challenge with a small number of macaques (*n* = 3) (16).

We hypothesized that by inducing mucosal Ab and T cell immunity, as well as innate immunity, the mucosal vaccine will be able to prevent or abort infection locally at the site of transmission before the virus disseminates systemically. This may be critical also because once the virus disseminates systemically, it can cause damage to other organs and widespread coagulopathies. To test this hypothesis, we developed and compared the immunogenicity and protective efficacy of 2 subunit vaccines, 1 systemic and 1 mucosal, in the rhesus macaque model. The systemic strategy is an intramuscularly (IM) administered vaccine composed of recombinant SARS-CoV-2 spike (S1) protein adjuvanted with alum. Subunit vaccines with alum have been traditionally used for vaccine development owing to their safety profile and effectiveness against viral infections (17–19). The mucosal strategy is a mucosal vaccine primed with IM-alum and boosted with IN-administered spike protein nanoparticles adjuvanted with TLR agonists and IL-15, analogous to vaccines we have used in HIV-1 studies (20–22). In light of the possibility that these vaccines could be used as a complementary booster vaccine after the administration of 2 doses of front-runner vaccines such as mRNA vaccines, adenovirus-vectored vaccines, or inactivated vaccines, we addressed the safety concerns and investigated the protective efficacy after 3 or 4 doses. The studies here indicate that after SARS-CoV-2 viral challenge, these 2 subunit vaccines could mediate full protection against viral replication in the upper and lower respiratory tracts, and interestingly, the IN vaccine could also clear the input challenge virus more rapidly to prevent viral transmission in the upper respiratory tract, which was only rarely achieved with most of the COVID-19 vaccine studies in macaques.

The adjuvanted subunit vaccines have important clinical implications considering the current situation. Several vaccines, including 2 mRNA strategies by Moderna and Pfizer, several adenovirus-vectored vaccines, and 2 inactivated vaccines, showed protection in phase III trials, and obtained or are close to obtaining license or Emergency Use Authorization (EUA). However, the durability of the protective immunity of these approaches is still unknown (23), so subsequent boosts may be necessary. Based on the previous experience with other coronaviruses, it is more likely that 1 or more additional boosts will be needed to induce long-lasting protective immunity after the waning of the induced Ab responses (24). Furthermore, recent emergence of new SARS-CoV-2 variants B.1.1.7 in the United Kingdom and B.1.351 in South Africa, which are more transmissible and the latter more resistant to convalescent plasma and vaccinee sera, calls for additional modified vaccine boosts (25, 26). Thus, safe and convenient booster vaccines, which could be given multiple times to humans, will likely be urgently needed in the future. Given the importance of respiratory mucosal immunity (12), our subunit mucosal vaccine could possibly be an ideal candidate to provide a potent and complementary reinforcement for any systemically induced immunity.

## Results

### Humoral responses after adjuvanted systemic and mucosal subunit vaccines.

**Figure 1:**
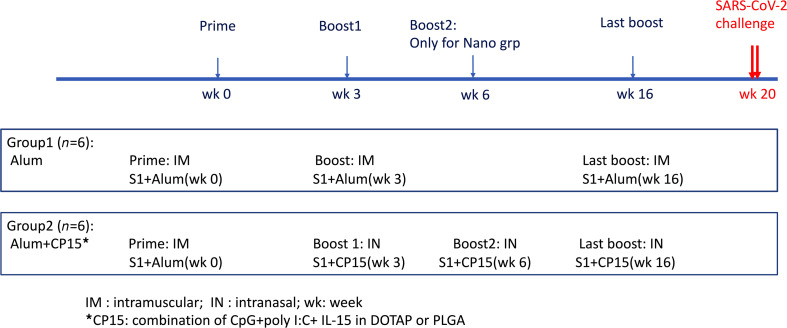
Schematic diagram of immunization protocol and groups.

Two groups of 6 Indian rhesus macaques each were included to test the immunogenicity of the 2 vaccine platforms. The systemic vaccine was IM primed and boosted with recombinant S1 protein in alum (group 1-alum group), whereas the mucosal vaccine was IM primed with S1 in alum, and IN boosted with S1-adjuvanted with a combination of IL-15 and TLR agonists (CpG and Poly I:C) incorporated in poly(lactic-co-glycolic acid) (PLGA) or 1,2-dioleoyl-3-trimethyl-ammonium-propane (DOTAP) nanoparticles (group 2-CP15 group). A total of 100 μg WT S1 protein per dose was used in both vaccines. The protein dose was chosen based on our previous HIV vaccine studies, whereas S1 protein was chosen because it is more immunogenic than receptor-binding domain (17) but has fewer other epitopes to compete than the full-length spike protein. All the animals were primed at week 0 and boosted at week 3 (Figure 1). An extra IN boost was given to group 2 at week 6. The first 2 IN boosts were in PLGA nanoparticles. Sixteen weeks after the first vaccination, 25 days before SARS-CoV-2 viral challenges, both groups 1 and 2 were boosted with S1 adjuvanted with either alum (IM) or CP15 in DOTAP nanoparticles (IN), respectively.

**Figure 2:**
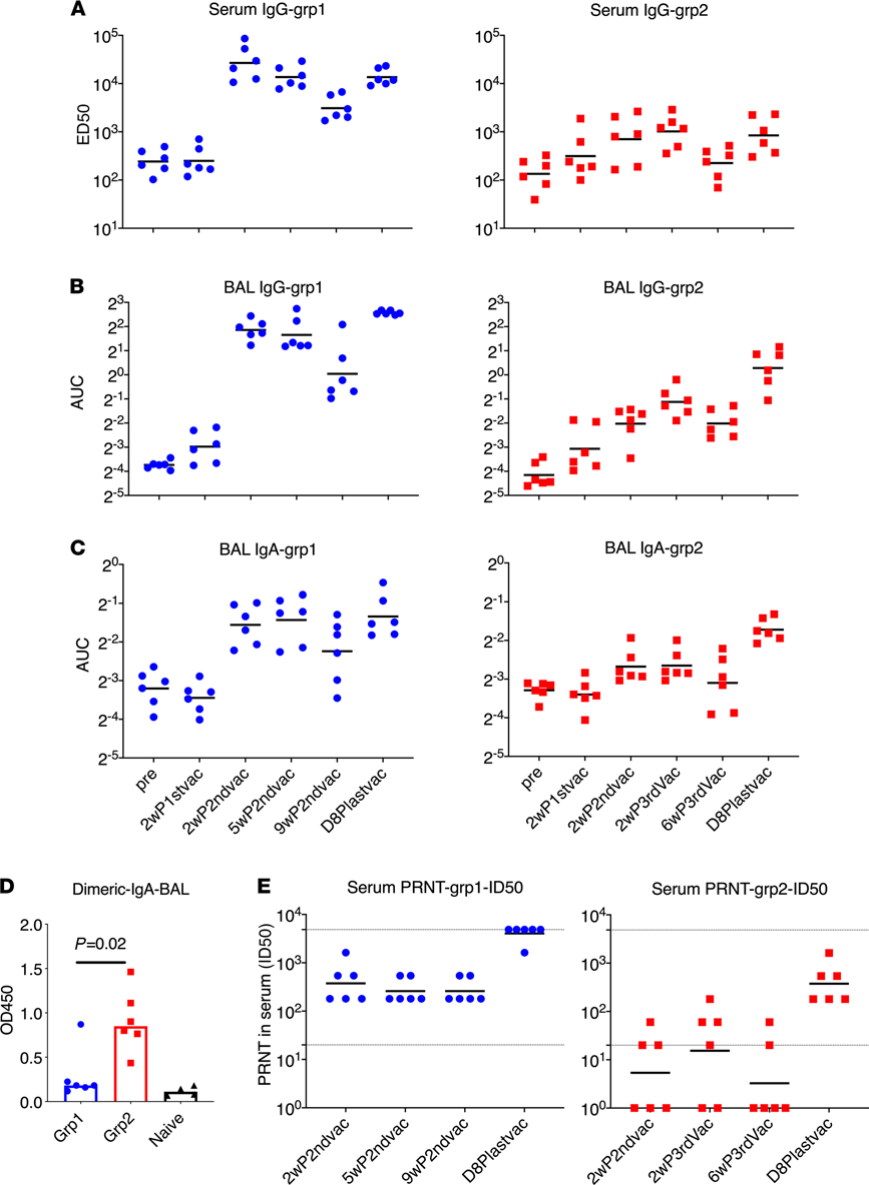
Spike-specific humoral immune responses in PBMC and bronchoalveolar lavage samples of the vaccinated animals. The ED_50_ of S1-specific IgG in serum (**A**) and the AUC of S1-specific IgG and IgA in bronchoalveolar lavage (BAL) (**B** and **C**) were measured during the whole course of vaccination. Dimeric IgA responses in BAL at day 8 after last vaccination (**D**) and PRNT (neutralizing) titers against live virus (**E**) in the serum samples were measured. BAL samples from naive animals (*n* = 4) were included (**D**) to serve as a negative control to show the baseline. The Mann-Whitney *U* test was used to assess the difference between groups 1 and 2 (**D**). Short lines show geometric means. Dashed lines show the lower and upper assay limits. *n* = 6 for group 1 and 2. 2wP2ndvac, 2 weeks after second vaccine dose; 5wP2ndvac, 5 weeks after second vaccine dose; 9wP2ndvac, 9 weeks after second vaccine dose; 2wP3rdvac, 2 weeks after third vaccine dose; 6wP3rdvac, 6 weeks after third vaccine dose; D8Plastvac, day 8 after last vaccination.

Because antibodies have been proposed to be the major protective mechanisms for most vaccine strategies (2, 4, 5), we first evaluated the S1-specific Ab responses in serum and bronchoalveolar lavage (BAL) fluid by ELISA (Figure 2, A–C). The first vaccination did not induce significant humoral responses over baseline in either platform. Two weeks after the second vaccination, robust S1-specific Ab responses, including serum IgG, BAL mucosal IgG and IgA, were elicited in group 1 animals, whereas much lower serum IgG and barely any BAL IgG and IgA responses were detected in group 2 animals (Figure 2, A–C). Group 1 reached a median serum ED_50_ of 25,209, whereas group 2 was significantly lower at 845 (Figure 2A). The IgG and IgA titers in BAL followed similar patterns (Figure 2, B and C). No significant boosting anamnestic effects were observed even with an extra IN boost at week 6 for group 2. In group 1, we observed declining Ab titers in serum and BAL over time, with about a 10-fold decrease of serum IgG titer (to 2596) at 9 weeks, compared with the peak at 2 weeks after the second vaccination.

Sixteen weeks after the first vaccination, an IM-alum booster dose was given to group 1 and an IN-CP15 booster dose in DOTAP was given to group 2 animals, leading to a significant anamnestic increase of serum IgG titer to 11,977 in group 1 and back to 824 in group 2 (Figure 2A). This last vaccination also resulted in the induction of mucosal IgG and IgA in BAL in group 2 (Figure 2, B and C). Nevertheless, after this boost, group 1 still had higher IgG responses in serum and BAL compared with those in group 2. Both groups had similar IgA responses in BAL. Dimeric IgA present at the mucosal surface has higher binding affinity to pathogens, and therefore is more potent than monomeric IgA, and thus may provide greater protection against mucosal pathogens (27, 28). We therefore assessed the S1-specific dimeric IgA responses in BAL samples. Notably, we found that group 2 had significantly higher dimeric IgA in BAL than group 1 (roughly 5-fold) after the last boost (Figure 2D). All but 1 animal in group 1 had only the same level of dimeric IgA as naive controls. This indicated that the total S1-specific IgA responses were different in the 2 groups, with group 2 having mainly dimeric IgA and group 1 having monomeric IgA. We hypothesized that the higher dimeric IgA responses in the lung mucosa of macaques receiving the mucosal vaccine might provide better protection against viral challenges with SARS-CoV-2 than the monomeric IgA responses.

All animals in group 1 had substantial neutralizing antibody (Nab) titers against live virus measured by plaque reduction neutralization test (PRNT) at 2 weeks after the second vaccination, whereas only 3 out of 6 animals had detectable Nab titers in group 2. The geometric mean titer (GMT) of Nab ID_50_ was 374 in group 1 and 18 in group 2 (Figure 2E). Interestingly, although the binding Ab titer in serum had a 10-fold decrease from 2–9 weeks after second vaccination, the PRNT titers maintained similar levels (Figure 2E). At day 8 after the last boost, even though the S1-binding Ab titer (11,977 and 824 for groups 1 and 2, respectively) was still lower than or comparable with that of the 2-week after second vaccination level (25,209 and 845, respectively), the ID_50_ of PRNT in group 1 (GMT greater than 4047) was so high that 5 out of 6 animals exceeded the upper detection limit of 4860. The GMT of PRNT in group 2 was also increased to 374. The ID_90_ of the PRNT data followed the same trend (Supplemental Figure 1; supplemental material available online with this article; https://doi.org/10.1172/jci.insight.148494DS1). Thus, the 2 platforms of S1 subunit vaccines induced robust S1-specific Ab responses in blood and BAL, including potent neutralizing capacity in blood. Based on the prior challenge studies using macaque models, protective effects were usually observed in animals with PRNT titers higher than 100 (1–5). The serum Nab titers of both our groups were higher than or comparable to those induced by other platforms tested in macaque models (1–5).

Notably, the last vaccination played a pivotal role in increasing the Nab titers for both groups. It is worth mentioning that since PLGA nanoparticles were hard to suspend, and therefore hard to administer IN, we switched to DOTAP nanoparticles for the last boost, which might partially account for the elevated humoral responses following the last IN dose in group 2. Although the mechanisms are not known, one hypothesis is that the interval of 2–3 months between the vaccination doses might give the Ab-producing B cells more time to interact with antigen-specific T helper cells and thus facilitate B cell maturation to high-affinity/Nab-producing plasma cells. Therefore, whether the vaccines could induce high-quality antigen-specific T helper cell responses was a key question.

### Cellular responses after adjuvanted systemic and mucosal subunit vaccines.

**Figure 3:**
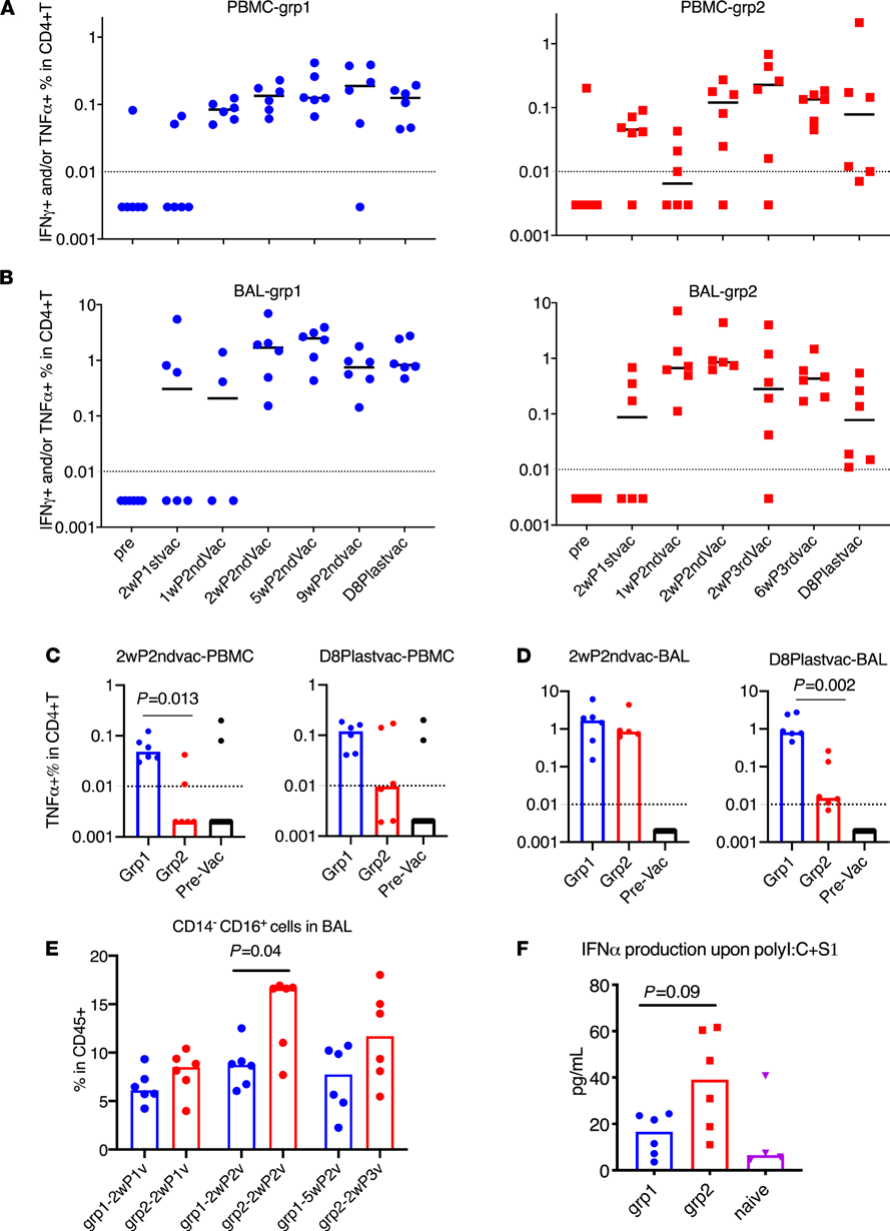
Spike-specific CD4^+^ T cell responses and trained immunity in PBMC and BAL samples of the vaccinated animals. Intracellular cytokine staining assays in responses to spike protein S1 were measured during the whole course of vaccination in PBMC (**A**) and BAL (**B**) samples. Spike-specific TNF-α^+^CD4^+^ T cell responses of different groups in PBMC (**C**) and BAL (**D**) samples at week 2 after second vaccination and day 8 after last vaccination were compared. PBMC and BAL samples from prevaccinated naive animals (*n* = 12) were included (**C** and **D**) to serve as negative control to show the baseline. The kinetics of CD14^–^CD16^+^ (monocyte or possibly NK) subsets were measured in the BAL samples of the vaccinated animals after 18 hours of PMA^+^ ionomycin stimulation (**E**). IFN-α was measured in the supernatant of BAL samples after 18 hours of Poly I:C plus S1 stimulation (**F**). Medians are shown. Serum from naive animals (*n* = 4) was included (**F**) to serve as negative control to show the baseline. Mann-Whitney *U* tests were used to compare the differences between groups (**D**–**F**). Dashed lines are the threshold for positive responses. *n* = 6 for groups 1 and 2.

Therefore, we evaluated the vaccine-induced S1-specific T cell responses throughout the whole course of vaccination. Even though the role of SARS-CoV-2–specific T cell responses in COVID-19 is still unclear, virus-specific CD4^+^ T cells can provide help for B cell activation and maturation and Ab induction (29–31). Th1 cell responses that secrete TNF-α and/or IFN are critical for this process. We measured different subsets of S1-specific T helper and CD8^+^ T cell responses in the PBMC and BAL samples of the vaccinated animals. Th1 cell responses were not induced until after the second vaccination. In both PBMCs and BAL, the dominant Th1 cell responses were TNF-α–secreting cells (Supplemental Figure 2). In group 1, the Th1 responses were persistent throughout the whole study in PBMC and BAL samples, whereas in group 2, the responses were durable in BAL but not in PBMC (Figure 3, A and B). Notably, group 1 animals had higher Th1 cell responses in the PBMCs than those in group 2 at both early and later time points during the vaccination sequence (Figure 3C). Similar Th1 cell responses in BAL were seen in both groups at early time points but dropped significantly in group 2 at later time points despite the mucosal immunizations that group 2 animals received (Figure 3D). Although not tested in this study, we speculated that the decrease might be attributed to the migration of the antigen-specific cells to the upper respiratory tracts after IN vaccination.

In other viral respiratory infections, including SARS-CoV and Middle East respiratory syndrome coronavirus, the presence of Th1 cell responses is more favorable to control disease, whereas the induction of Th2 and Th17 cell responses has been linked to immunopathogenic lung diseases in animals or clinical trials (32–34). When evaluating S1-specific Th2 (IL-4–, IL-13–secreting cells) and Th17 (IL-17A–secreting cells) responses, we did not find significant differences between the 2 vaccinated groups after the vaccination or in the prevaccination levels (Supplemental Figure 3). However, since the frequencies of antigen-specific T cell responses were low, we further assessed the kinetics of total Th1, Th2, and Th17 subsets after stimulating the samples with PMA and ionomycin. In these more robust assays, we observed a slight downtrend of Th1, an uptrend of Th17, and no change for Th2 in PBMC (Supplemental Figure 4). This was in sharp contrast to the scenario in BAL, where Th1 response increased over time, and especially the frequency of TNF-α–secreting cells was almost doubled compared with prevaccination levels (from 40% to 80%) (Supplemental Figure 4). Total TNF-α–secreting CD8^+^ T cells (Tc1) also increased markedly from 60% to 85% after stimulation with PMA and ionomycin (Supplemental Figure 5). This increase of Th1 and Tc1 responses in BAL for both vaccine platforms suggested that a redistribution of the Th cell and CD8^+^ T subsets might occur during the vaccination. The high frequency of Th1 and Tc1 subsets in BAL might be beneficial to the host, suggesting a further benefit of the local respiratory mucosal route of vaccination. The S1-specific CD8^+^ T cell responses were also induced in some of the vaccinated animals from both groups but with less magnitude and persistence (Supplemental Figure 6).

We have used a similar platform with TLR agonists plus IL-15 as adjuvants to develop an HIV vaccine, in which trained innate immunity was induced and was involved in mediating protection against viral transmission (21, 35). Trained immunity is characterized by enhanced innate responses after encounter with the pathogens the second time, and this is usually achieved through epigenetic modification of genes in myeloid or natural killer cells (36–38). In this study, we first measured the frequency of changes of CD14^+^ and/or CD16^+^ populations in BAL. Interestingly, the CD14^–^CD16^+^ population showed a significant increase in group 2 compared with those of group 1 two weeks after receiving the second vaccination (*P* = 0.04) and also increased compared with samples before receiving the CP15 adjuvants (*P* = 0.002; Figure 3E). However, more boosting (third vaccination) did not further increase the frequency of these cells (Figure 3E). Owing to the lack of cell markers, we cannot distinguish whether these cells were myeloid cells or NK cells.

We next measured the IFN-α expression levels in BAL samples after exposure to the viral mimic: Poly I:C plus S1 protein. BAL samples collected at a later time point would be a better marker than the early ones. However, because the small BAL samples collected at later time points were used up for antigen-specific T cell responses, we had to use 1 week after vaccination BAL samples to measure IFN-α expression. Upon stimulation with Poly I:C and S1 protein ex vivo, the BAL samples from group 2 produced higher levels of IFN-α in the supernatant than those of group 1 or the naive group (Figure 3F), whereas other cytokines and chemokines did not differ significantly between the groups (Supplemental Figure 7). These data suggested that trained innate immunity, represented by the CD14^–^CD16^+^ subpopulation and the production of IFN-α upon stimulation, was induced by S1 with CP15 adjuvant (CpG, Poly I:C plus IL-15).

### Viral load in nasal swab and BAL samples after IN and intratracheal routes of SARS-CoV-2 viral inoculations.

To test the vaccine efficacy, about 4 weeks after the last vaccination, we challenged the 12 vaccinated and 6 naive macaques with 1.5 × 10^4^ PFU SARS-CoV-2 virus (USA-WA1/2020 strain), which was equivalent to approximately 1.25 × 10^5^ tissue culture infectious dose 50 (TCID_50_). The challenge virus was obtained from BEI Resources and has a reported infectious titer in Vero E6 cells of 3 × 10^6^ PFU/mL. The dose was chosen to be approximately the same (1.1 × 10^4^ PFU) as the dose established by the 2 published studies carried out at the same facility (BIOQUAL Inc.) (2, 5). The animals were challenged via both IN and intratracheal routes in order to deliver the virus to both upper and lower airways simultaneously. Genomic RNA (gRNA) and subgenomic RNA (sgRNA) PCRs were performed to quantify the input and replicating virus respectively (39, 40). SgRNA in particular is an indication of replicating virus.

**Figure 4:**
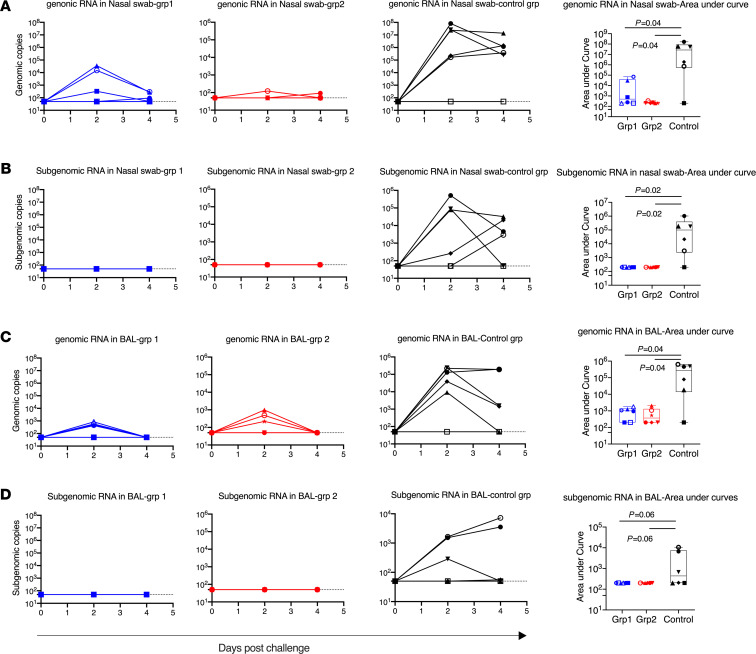
Viral load in nasal swabs and BAL fluids after SARS-CoV-2 intranasal/intratracheal challenges. SARS-CoV-2 genomic RNA and subgenomic RNA were assessed in the nasal swabs (**A** and **B**) BAL fluid (**C** and **D**) collected at days 2 and 4 after viral challenges. AUC was calculated for each animal and plotted in the box and whisker plots, where the median, other quartiles, and minimum to maximum are shown. The assay lower limit (50 copies) is shown as dashed lines. In each panel, Mann-Whitney *U* tests corrected for multiple comparisons by the Hochberg method were used to compare the viral load AUC differences between vaccinated groups and the SARS-CoV-2 naive control group. *n* = 6 for group 1, group 2, and naive group. Each animal has a unique symbol with different shape and color, which is consistent throughout (**A**–**D**).

After viral challenge, 5 out of 6 SARS-CoV-2–naive control animals demonstrated clear signs of viral replication, shown by sgRNA viral load (VL). Among the 5 infected animals, 3 animals had viral replication in both nasal swabs and BAL fluid, and 2 animals had sgRNA in nasal swabs but not in BAL fluid (Figure 4). Similar to other studies of SARS-CoV-2 vaccines in macaque models, the input VLs were much higher than the replicating VLs. At day 2, a VL of log 7 in nasal swabs and a VL of log 5 in BAL fluid were detected. One animal, DFKL, in the naive group, did not shown any signs of infection. Even the input virus, as shown in gRNA VL, was negative in all samples tested. It is worth mentioning that DFKL previously had been exposed to 8 repeated challenges of SIVmac251, but never showed any VLs for SIV, suggesting that this animal might have unique innate immunity, which allowed it to quickly clear the input virus. Indeed, we found that this animal had unusually high levels of IFN-α, SCF, I-TAC, IL-1R-α, and PDGF-BB in serum. The high level of IFN (undetectable in naive uninfected samples) might explain the resistance of DFKL to SIVmac251 and SARS-CoV-2 viral challenges (Supplemental Figure 8).

In the vaccinated groups, through the whole course of infection, we did not detect any sgRNA in the nasal swabs and lung fluid of any animals (Figure 4, B and D). These data suggest that both vaccine platforms mediated 100% protection against replicating virus in both tissues, which has been rarely seen with previous COVID-19 vaccines in macaques. Even the input virus gRNA was rapidly cleared in the nasal swabs of 3 of 6 in group 1 and 5 of 6 in group 2 animals already at day 2 after infection. In the BAL fluid, the input virus was also cleared in 2 group 1 and 3 group 2 animals at day 2, and all were cleared by day 4 (Figure 4, A and C).

### Immune correlates after vaccination and viral challenges.

**Figure 5:**
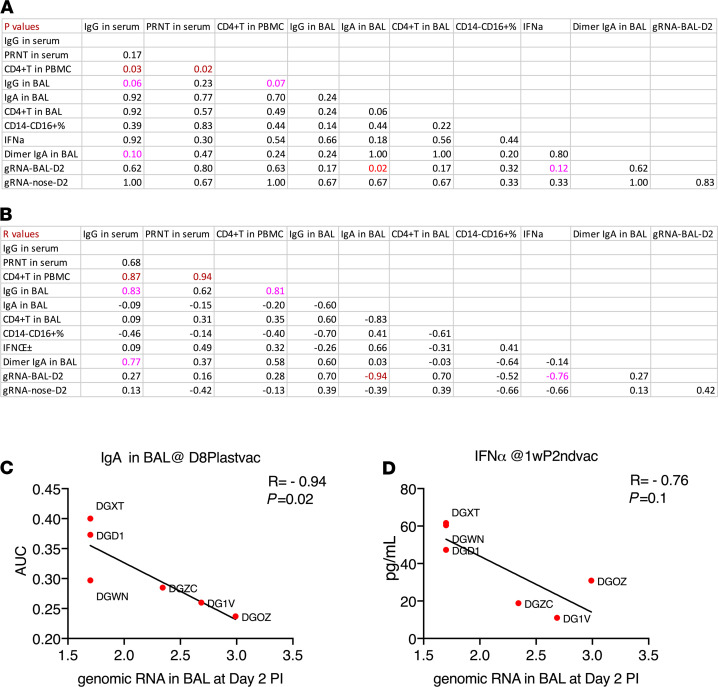
Immune correlations after vaccination and viral challenges in group 2. The *P* (**A**) and *R* values (**B**) of the immune correlation matrix among antigen-specific humoral, cellular responses, innate immunity, and genomic RNA in BAL at day 2. The peripheral and BAL samples were collected at day 8 after last vaccination or early time points (noted). Prechallenge IgA titer in BAL (**C**) and IFN-α production in ex vivo–stimulated BAL cells (**D**) were correlated with day 2 postchallenge genomic RNA in BAL. Spearman’s *R* and *P* values are shown. *n* = 6 for group 2.

Since full immunity against sgRNA had been achieved for both vaccines, we could not identify the immune correlates of protection at the sgRNA level. However, we further analyzed the immune correlates with peak gRNA data after the mucosal vaccine, which is a surrogate marker of efficiency of clearance of input virus. Since group 1 and 2 animals had different immune responses and might have different protection mechanisms, it was more logical to analyze them separately in order to have the capability to compare between the 2 groups. Since most of the immune responses in group 1 were very similar to each other, there was not enough spread to find significant correlations within that group (data not shown). We did observe several significant correlations or trends of significance in group 2 (Figure 5, A and B). Notably, both serum S1-specific IgG and PRNT responses positively correlated with antigen-specific CD4^+^ T cell responses in PBMCs (*R* = 0.94 and 0.87; *P* = 0.02 and 0.03, respectively), suggesting the importance of antigen-specific Th1 responses to induce humoral responses. Importantly, we noticed that gRNA in BAL inversely correlated (or showed a trend) with S1-specific IgA titers and IFN-α production in BAL samples (Figure 5, C and D, *R* = –0.94 and –0.76; *P* = 0.02 and 0.12, respectively), suggesting that local respiratory mucosal immunity might participate in clearing of the input virus more efficiently (Figure 5). We did not find a correlation between dimeric IgA and gRNA clearance after viral challenge. One possible explanation is that whereas dimeric IgA, as an ideal mucosal defender, can efficiently neutralize virus by immune exclusion to prevent the virus from contacting epithelial cells, or trapping the invaders on the luminal surface, dimeric IgA is a poor opsonin and a weaker activator of complement system and thus is not capable of clearing the virus-Ab complexes as quickly as IgA does. However, the higher dimeric IgA titers in group 2 (Figure 2D) may contribute to inhibiting viral replication by preventing the virus from infecting the target cells. Thus, both mechanisms may play a role and are worth further investigation.

### Histopathology after viral infection.

**Figure 6:**
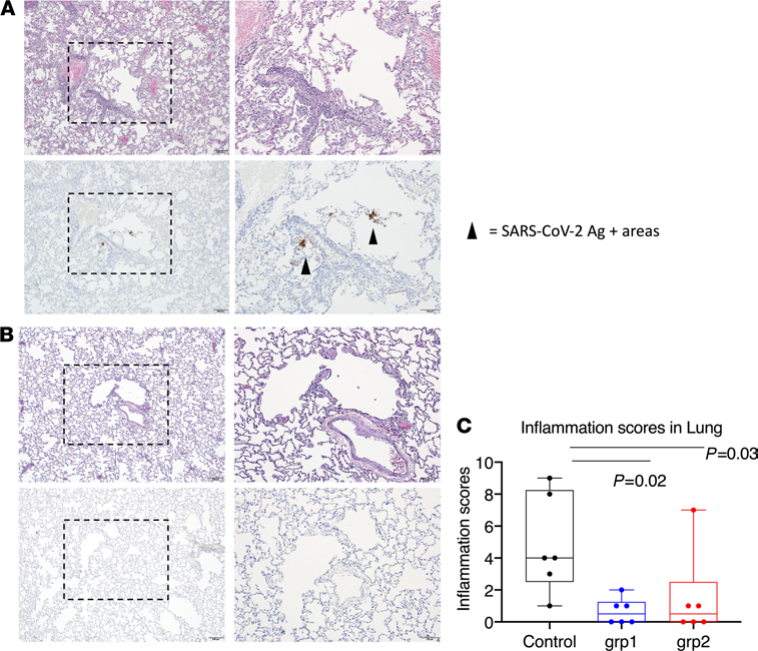
Histopathological analysis and viral antigen detection in the lung. Seven or 10 days after challenge, lungs were harvested, and multiple sections of lung were evaluated histologically and immunohistochemically for the presence of SARS-CoV-2–related inflammation and SARS-CoV-2 virus antigen. Representative images were from lungs harvested day 7 from 1 animal in the naive group (**A**) and 1 animal in vaccinated group 1 (**B**). Each animal was blindly scored by a pathologist based on the degree of inflammation in the lung. In the box and whiskers plot, the median, other quartiles, and minimum and maximum are shown. Mann-Whitney *U* tests corrected for multiple comparisons by the Hochberg method were used to compare the lung inflammation between SARS-CoV-2 naive control and vaccination groups (**C**). Scale bars: 200 μm (original magnification, ×4) and 100 μm (original magnification, ×10). *n* = 6 for groups 1 and 2 and naive group.

Throughout the study, we did not observe any clinical abnormalities in the control and study group animals. As there were not enough staff to have all the animals necropsied in 1 day, we performed the necropsies on either day 7 or 10 (Supplemental Table 1). One-half of the animals in each group were euthanized on day 7 and the other half on day 10. The distribution is evenly divided, and therefore, the histopathology results/lung inflammation scores are comparable. The timing was also dependent on the need to first collect BAL fluid on days 2 and 4 after challenge. Sections of lung and lymph node (axillary and inguinal) from animals necropsied on day 7 were evaluated histologically and immunohistochemically for the presence of SARS-CoV-2–associated inflammation and SARS-CoV-2 virus antigen, respectively. Most lung sections were negative for virus antigen immunoreactivity, but in some cases, rare positive foci of virus antigen were observed in samples from 2 control animals (Figure 6, A and B). The severity of inflammation, when present, ranged from mild to moderate severity. The inflammatory changes observed were characterized by a mixed polymorphonuclear and mononuclear (predominantly macrophage) cellular infiltrate present within alveolar capillaries and, less frequently, present within the alveolar spaces. Inflammatory lesions were most associated with regions surrounding small bronchioles and small-caliber blood vessels. Perivascular infiltrates were largely composed of small lymphocytes and fewer histiocytes. Significant inflammation was largely absent in the sections of lung examined for this cohort. Each animal was given an inflammation score based on the evaluation of lung infiltration (Supplemental Table 1). In accordance with the VL data, the scores from the SARS-CoV-2 naive control group were significantly higher than those from the vaccinated groups (Figure 6C). There was no evidence of significant inflammation or virus antigen observed in the sections of lymph node examined. The 2 naive animals that showed positive virus antigen staining in the lung had the highest gRNA VL and highest inflammation scores, consistent with the fact that the inflammation was induced by viral infection. However, we also observed prominent lung inflammation from 1 vaccinated animal from group 2, which did not show any gRNA or sgRNA in either nasal swabs or BAL at any time points tested, suggesting the inflammation was sometimes induced by factors other than viral infection. Interestingly, the only animal that did not become infected in the naive group also demonstrated a certain level of inflammation in the lung (Supplemental Table 1).

## Discussion

We have developed 2 vaccine platforms that we have shown here to be 100% protective against SARS-CoV-2 viral replication (free of sgRNA), which has been only rarely achieved in macaque studies (1–5, 41). Furthermore, the mucosal vaccine seems more efficient at rapidly clearing the input virus (gRNA) in the upper respiratory tract than the systemic counterpart, providing a potent strategy to prevent viral transmission. However, since the protection against sgRNA was so complete, we were not able to assess the potential immune correlates for the full immunity against replicating sgRNA, but we did find 2 correlates of clearance of input challenge virus — BAL IgA and IFN-α, induced by the mucosal immunization. Different animal models like hamsters or ferrets, which are more sensitive to viral transmission and disease, might help to identify the immune correlates of protection in the future studies. Indeed, in a recent study using the hamster model, we observed that the IN mucosal vaccine mediated significant protection against SARS-CoV-2 challenge, whereas the systemic vaccine only showed a trend of significant protection compared with naive controls (our unpublished observations).

The mucosal vaccine (CP15-IN) is of particular interest in that it mediated full protection in both the lower lung and nasal cavity with relatively low Nab titers, implying complementary additional protective mechanisms. Even though our early Nab titers were comparable (Alum-IM) or not as good (CP15-IN) as those of an mRNA vaccine (4), the last boost increased the Nab titers higher (Alum-IM) or to a level (CP15-IN) comparable to that of the mRNA vaccine. Compared with macaques vaccinated with 10 and 100 μg mRNA-1273, which induced Nab titers of 501 and 3481, respectively (4), after the last boost, the mucosal vaccine described here induced a lower Nab titer of 374. Yet, our mucosal vaccine demonstrated outstanding protection in both upper and low respiratory tracts. In the nasal cavity, 0 of 6 animals had detectable viral sgRNAs, a measure of viable replicating virus, and only 1 of 6 animals had detectable viral gRNAs, a measure of residual challenge virus, 2 days after viral challenge. With the systemic vaccine, which induced a much higher Nab titer (more than 4047), 3 of 6 animals had gRNA in their nasal swabs. Therefore, although we cannot identify the exact mechanisms of protection for the mucosal vaccine, Nab titer cannot be the only protective mechanism for virus clearance, and other mechanisms should be examined in future COVID-19 vaccine trials.

Two parameters might account for the complete protection against viral replication (sgRNA) by the mucosal vaccine without high titers of Nab or T cell responses. The mucosal vaccine induced a qualitatively different response in the lung, with more dimeric IgA compared with monomeric IgA. This qualitative difference might outweigh the total quantity of IgA or IgG measured. Another parameter was the higher frequency of CD14^–^CD16^+^ cells in the lung after boosting via the mucosal route, which was associated with higher production of IFN-α upon restimulation with a viral infection mimic (S1 protein + Poly I:C dsRNA). IFN-α and/or dimeric IgA may be critical for the mucosally vaccinated animals to control viral replication and rapidly clear input virus, especially at the mucosal surface. This is consistent with reports that low IFN-α in human patients correlates with more severe COVID-19 disease (42), and inborn defects in type I IFN or autoantibody against type I IFN leads to life-threatening COVID-19 disease (43, 44). Thus, these results suggest that the qualitatively different responses induced in the lung by the mucosal vaccine boosts may be valuable to complement immunity induced by conventional systemic vaccines against respiratory virus transmission.

However, there were limitations in the study design. Since we proposed the use of the subunit vaccine as a potent reinforcing IN boost, it is ideal to give the IN boost after the conventional EUA-granted systemic vaccines, such as mRNA vaccines and adenovirus-vectored vaccines, which may be a future direction. Nevertheless, group 2 animals received an S1 prime in alum followed by mucosal boosts with nanoparticles, so the systemic prime and mucosal boost strategy was indeed tested here. Moreover, control animals should include naive animals, as well as placebo animals receiving a proper placebo, e.g., irrelevant protein with adjuvant. However, owing to the lack of rhesus macaques available in the market, in this study, we only have SARS-CoV-2–naive control animals that have been exposed previously to HIV-1 envelope protein/peptides (which are irrelevant proteins to SARS-CoV-2) and adjuvant alum (one of the adjuvants used in this study), but not exposed to another adjuvant CP15.

Licensing or EUA of 2 mRNA vaccines, several adenovirus-vectored vaccines, and inactivated vaccines was granted in multiple countries for administration after protective efficacy was demonstrated. These vaccines are safe with 1 or 2 doses. However, the durability of the protective immunity of these vaccines is still unknown and may need more boosts in the future. The newly emerging SARS-CoV-2 variants, which could escape the vaccine- or infection-induced neutralizing activity, may reduce the vaccine efficacy. Thus, booster vaccines, which will be administrated as a third or more doses, are urgently needed as well. Heterologous boosts may be more effective. Here, we demonstrated as a proof of concept that the adjuvanted subunit vaccines serve as an ideal booster candidate, especially with the mucosal nanoparticle delivery. Both vaccines appear safe, and we did not observe any vaccine-induced immune pathology even after 3 or 4 doses. Most importantly, we demonstrated in the macaque model that the third or fourth doses of adjuvanted subunit vaccine mediated full protection against viral challenges. Specifically, the mucosal boost induced local respiratory mucosal protection and potently complemented or synergized with systemic immunity to quickly clear the virus in the nasal cavity, preventing viral transmission. The ability of vaccines to prevent transmission is an important concern from a public health standpoint. Local respiratory mucosal immunity that can clear the virus inoculum at the site of transmission before it disseminates systemically could also potentially prevent serious complications of COVID-19, such as blood clotting disorders and kidney, heart, liver and brain damage. Although our approach is early in preclinical testing, we believe that it may provide a novel strategy to boost local vaccine immunity for the next generation of SARS-CoV-2 vaccines.

## Methods

### Animals.

Eighteen Indian-origin adult male rhesus macaques (*Macaca mulatta*), 3–8 years old, were included in the study. At the start of the study, all animals were free of cercopithecine herpesvirus 1, SIV, simian type-D retrovirus, and simian T lymphotropic virus type 1.

### Study design for subunit vaccine with adjuvants.

No animals had been exposed to SARS-CoV-2 prior to challenge, and all tested negative for SARS-CoV-2 before the study. Six macaques were included in the SARS-CoV-2–naive control group and had previously gone through HIV envelope protein/glycopeptide vaccination, and one of them (DFKL) had been exposed to 8 repeated challenges of SIVmac251 but never showed any VLs for SIV. An additional 12 macaques that were never enrolled in any other studies were divided into 2 vaccine groups. Group 1 (*n* = 6, alum group) was given systemic vaccine primed at week 0 and boosted at weeks 3 and 16 with SARS-CoV-2 S1 protein with alum adjuvant. All the vaccinations were given IM in group 1. Group 2 (*n* = 6, CP15 group) was administered with a mucosal vaccine primed at week 0 with S1 protein with alum adjuvant (administrated IM), and boosted at weeks 3, 6, and 16 with S1 protein with CP15 adjuvant (administrated IN), which was a combination of CpG + Poly I:C + IL-15 in DOTAP or PLGA. For immunization, each vaccine contained 100 μg of recombinant SARS-CoV-2 (2019-nCoV) spike S1 protein (catalog 40591-V08H, Sino Biological, endotoxin level: <0.001 U/μg). A total of 100 μL Adju-Phos adjuvant (aluminum phosphate gel, InvivoGen) was used as adjuvant for IM administration in a 1 mL volume. CP15 adjuvant was a combination of 200 μg per dose of D-type CpG oligodeoxynucleotide, 1 mg per dose of Poly I:C (InvivoGen), and 200 μg per dose of recombinant human IL-15 (Sino Biological). The mucosal vaccine incorporated S1 protein with CP15, formulated in nanoparticles either in PLGA (Alchem Laboratories Corporation) for the first 2 doses or in DOTAP (100 μL per dose; Roche) for the last dose. CP15 adjuvanted mucosal vaccine was given IN in a volume of 100 μL per nostril, whereas the animals were anesthetized. After vaccination, blood and BAL fluid samples were collected at the times noted and analyzed.

### BAL sample collection.

Animals were anesthetized, and then up to 10 mL/kg sterile saline was instilled into the lungs. The instilled fluid (up to 90%) was recovered by suction. A 100 μm cell strainer was used to remove large pieces from the collected BAL fluid. The cells were then washed with R10 medium (RPMI-1640 with 10% FBS) and centrifuged at 1500 rpm, for 5 minutes at room temperature. BAL fluid and cells were collected for analysis or cryopreservation.

### ELISA to detect S1-specific Ab responses.

The BAL samples collected from each individual monkey were concentrated roughly 30-fold using Amicon Ultra centrifugal filter units (10 kDa cutoff, MilliporeSigma). The total IgA quantity in the concentrated BAL samples was determined using the Monkey IgA ELISA development kit (HRP) (Mabtech) following the manufacturer’s protocol.

Total IgG quantities in the plasma and concentrated BAL samples were measured using the Rhesus Monkey IgG-UNLB (Southern Biotech) as the IgG standard. Briefly, high-binding 96-well plates (Santa Cruz Biotechnology) were coated with serial dilutions of IgG standard and the samples in 1× PBS, pH 7.4, and incubated at 4°C overnight. Afterward, the plates were washed 3 times with wash buffer (0.05% Tween-20 in 1× PBS, pH 7.4) and blocked with 300 μL 2% sodium casein in 1× PBS at 37°C for 1 hour. Following 3 washes, 100 μL goat anti-monkey IgG (H+L) Secondary Ab [HRP] (Novus Biologicals catalog: NB7215) was applied to each well with 1:20,000 dilution in 1× PBS. The plates were incubated at room temperature for 30 minutes and then extensively washed with the wash buffer 5 times. Then, 3,3′,5,5′-tetramethylbenzidine (TMB) 2-component microwell peroxidase substrates (SeraCare) were applied to the well plates following the manufacturer’s instructions. The plates were developed in the dark at room temperature for 30 minutes and then quenched by adding 100 μL/well 1 M H_3_PO_4_ solution. Absorbance was read using SpectraMax M5 Multi-Mode Microplate Reader (Molecular Devices) at 450 and 550 nm. The concentrations of IgA and IgG were determined using GraphPad Prism 8 software with sigmoidal nonlinear regression.

The antigen-specific binding assays were performed similarly but with 100 ng/well SARS-CoV-2 spike S1-His Recombinant Protein (Sino Biological) as the coating antigen. After blocking the plates with 2% sodium casein, the concentrated BAL samples were applied in duplicate with a series of 2-fold dilutions, starting from an IgA or IgG concentration of 2 μg/mL. In the case of antiserum analysis, plasma samples were serially diluted 2-/4-/5-fold starting from a 1:150 dilution and run in duplicate. The plates were incubated at room temperature for 1 hour, followed by 4 washes. Subsequent steps of incubation with HRP-labeled secondary Ab and TMB substrate were followed as previously described. In case of BAL IgA binding assay, goat anti-monkey IgA (α chain specific) HRP conjugate (1:5000 dilution, Alpha Diagnostic catalog: 70041) was used as a secondary Ab. After assay, AUC, endpoint titer, and ED_50_ values were computed by GraphPad Prism 8 software with sigmoidal nonlinear regression.

### ELISA to detect dimeric IgA in BAL.

DuoSet ELISA Ancillary Reagent Kit 2 (R&D Systems, Bio-Techne) was used. Briefly, 100 ng/well SARS-CoV-2 spike S1 protein was coated and blocked as previously described. Original BAL samples from vaccinated and naive animals were added in duplicate to the plate and incubated at room temperature for 1 hour, followed by 5 washes. Mouse anti-rhesus J chain (CA1L_33e1_A1a3) Ab (1:1000 dilution, NIH Nonhuman Primate Reagent Resource) and goat anti-mouse IgG-HRP conjugate (1:10,000 dilution, R&D Systems, Bio-Techne, catalog: HAF007) were added and each followed by 1 hour of incubation at room temperature and 5 washes. Plate development and reading was performed as previously described.

### PRNT.

PRNT was performed in duplicate using Vero E6 cells (ATCC, catalog CRL-1586) and 30 PFU challenge titers of SARS-CoV-2 (USA-WA1/2020 strain) (45). Serum samples were tested at a starting dilution of 1:20 and were serially diluted 3-fold up to final dilution of 1:4860. After serum incubation with 30 PFU of SARS-CoV-2 for 1 hour at 37°C, serial dilutions of virus-serum mixtures were added onto Vero E6 cell monolayers. Cell culture medium with 1% agarose was added to the cells, following incubation for 1 hour at 37°C with 5% CO_2_. The plates were fixed and stained after 3 days of culture. Ab titer ID_50_ and ID_90_ were defined as the highest serum dilution resulting in 50% and 90% reduction of plaques, respectively.

### Intracellular cytokine staining assay.

SARS-CoV-2–specific T cells were measured from mononuclear cells of the fresh or thawed cryopreserved BAL and PBMC samples by flow cytometric intracellular cytokine analysis, as previously described in detail (20, 46). Briefly, cell samples were stimulated with 2 μg/mL SARS-CoV-2 S1 protein (Sino Biological) for PBMC, and 5 μg/mL for BAL, samples with 0.15 μg/mL brefeldin A at 37°C 5% CO_2_ overnight. Negative controls received an equal concentration of brefeldin A (without protein). Cell activation cocktail with PMA (20.25 pM) and ionomycin (335 pM) and 0.15 μg/mL brefeldin A (BioLegend) was added to the cells as positive control. For flow cytometric analysis, the BAL cells were centrifuged after a wash with 0.25% PBS and then stained with viability dye (Invitrogen, Thermo Fisher Scientific) and Ab mixtures. Abs PE-Cy7-CD3, BV605-CD4, APC-Cy7-CD8, and Alexa Fluor 700–CD45 were from BD Biosciences; FITC-CD28, Pe-Cy5-CD95, BV711–TNF-α, IFN-γ–PE or –PerCP, Alexa Fluor 647–IL-4, BV785-IL-2, BV421–IL-17A, BV785-CD14, and BV421-CD16 were from BioLegend; and PE–IL-13 was from Miltenyi Biotec. Detailed Ab information is listed in Supplemental Table 2. After cell surface staining, eBioscienceFOXP3/Transcription Factor Staining Buffer Set (Thermo Fisher Scientific) was used for cell permeabilization, followed by intracellular staining. An LSRII flow cytometer with 4 lasers (BD Biosciences) and FlowJo software (BD) was used for data acquisition and analyses. For each animal, and each time point, the antigen-specific T cell responses were reported as the frequencies of cytokine-positive cells in the samples stimulated with S1 protein minus those in the medium-only control.

### IFN-α ELISA and chemokine/cytokine Bioplex assay after Poly I:C plus S1 protein stimulation of BAL samples.

Cryopreserved BAL samples (from the 1 week after second vaccination time point) were thawed and resuspended at a concentration of 3–4 million cells/mL in serum-free medium AIM V (Thermo Fisher Scientific). Poly I:C (2 μg/mL) was added to the cells in the presence or absence of 2 μg/mL of SARS-CoV-2 spike S1 protein (Sino Biological; endotoxin level: <0.001 U/μg). After 18 hours of culture at 37°C, 5% CO_2_, supernatant was collected and frozen at –20°C for IFN-α ELISA and Chemokine/Cytokine Bioplex Assay using an LSRII cytometer. LEGENDplex NHP Chemokine/Cytokine Panel (13-plex, BioLegend) was used to measure the following 13 chemokines and cytokines: TNF-α, IL-1β, IL-6, IL-8, MIP1-α, MIP1-β, RANTES, MCP-1, IFN-γ, MIG, IP-10, ITAC, and Eotaxin. Pan–IFN-α (including subtypes α1, 2, 4, 5, 6, 7, 8, 10, 14, 16, and 17) ELISA kit (Mabtech) was used to measure the total concentration of IFN-α. Both assays were performed in accordance with the manufacturers’ instructions.

### SARS-CoV-2 challenge.

At week 20, 25 days after the last boost, all 18 animals were challenged with 1.5 × 10^4^ PFU SARS-CoV-2 (USA-WA1/2020 strain), which was equivalent to approximately 1.25 × 10^5^ TCID_50_ SARS-CoV-2 virus (USA-WA1/2020 strain), equivalent to or slightly greater than the challenge dose used in some earlier macaque challenge studies noted above. The challenge virus was obtained from BEI Resources (lot 70038893) and has a reported infectious titer in Vero E6 cells of 3 × 10^6^ PFU/mL. The virus was diluted in PBS to the indicated challenge dose level. The virus was given IN and intratracheally, each route with 1 mL (0.5 mL for each nare) to make sure the virus was delivered to both the upper and lower airway. Nasal swab and BAL fluid samples were collected on days 2 and 4 after challenge to measure the VL.

### sgRNA and viral RNA assay.

SARS-CoV-2 RNA levels were monitored by reverse transcription PCR by BIOQUAL Inc. as previously described (5). Briefly, RNA was extracted from nasal swab and BAL fluid samples collected at the different time points. After reverse transcription, cDNAs were run in duplicate to quantify subgenomic or viral RNA using different primer/probe sets, targeting the viral E gene mRNA or the viral nucleocapsid, respectively. The sequences of the primers/probes have been published previously (5, 40). VLs are shown as copies/mL for BAL fluid and per swab for nasal samples with a cutoff value of 50 copies for each assay.

### Lower respiratory histopathology and immunohistochemistry.

Seven or 10 days after SARS-CoV-2 viral challenge (when VLs on BAL specimens from day 4 could be obtained and because it was not feasible to necropsy all on the same day), one-half of the animals from each group were necropsied on each day, and the lower respiratory (lung) tissue specimens were collected, fixed, processed, and embedded in paraffin blocks and sectioned at a thickness of 5 μm. Immunohistochemistry was used to study sections from animals necropsied at day 7. The sections were stained with H&E and examined by light microscopy. Multiple sections of lung and lymph node (axillary and inguinal) were evaluated histologically and immunohistochemically for the presence of SARS-CoV-2–related inflammation and SARS-CoV-2 virus antigen, respectively. A rabbit polyclonal SARS-CoV-2 Ab (GeneTex) was used for immunohistochemical staining.

The inflammatory cellular constituents were largely similar for all groups where inflammation was observed (mixed polymorphonuclear and mononuclear cells), so severity is based on percentage tissue affected and the presence or absence of other indicators of inflammation and tissue damage (fibrin/edema/luminal debris/hemorrhage/necrosis). In addition to lesion severity, lesion distribution and the location were recorded; lesions were either associated with/exhibited as alveolar interstitium (Alv) changes, intra-alveolar infiltrates (intraAlv), changes associated with bronchi (Br) or bronchioles (br), or perivascular spaces (PV) or exhibited variable degrees of type II pneumocyte hyperplasia (type II). Inflammation in the lung was scored using the following severity scale: normal = – (0); <10% (tissue affected) = +/– (1); >10 to <25% = + (2); >26 to <50% = ++ (3); and >50% = +++ (4). Three parts of the lung (left caudal, right middle, and right caudal lobes) were evaluated and scored by a board-certified veterinary pathologist, who was blind to the groups. The total inflammation score was calculated as the sum of the 3 parts. Sections were evaluated using an Olympus BX51 bright-field microscope, and representative photomicrographs were captured using an Olympus DP73 camera.

### Statistics.

Statistical analyses were performed using Prism version 8 (GraphPad). Mann-Whitney *U* and Wilcoxon tests were used for group comparisons with Benjamini-Hochberg corrections for multiple comparisons where appropriate, and Spearman’s analyses were used for correlations, as shown in the figures. All statistical tests were 2 tailed. A *P* value of less than 0.05 was considered significant.

### Study approval.

All animals were initially housed at the NIH National Cancer Institute (NCI) Animal Facility for vaccination. The NIH is an American Association for the Accreditation of Laboratory Animal Care–accredited facility and has a PHS Approved Animal Welfare Assurance (Assurance ID A4149-01). All the animal studies were approved (under protocol VB-037) by the NCI IACUC. Two weeks before challenge, the animals were moved to a qualified BSL3 biohazard facility at BIOQUAL Inc. for SARS-CoV-2 viral challenge study (Rockville, Maryland, USA). BIOQUAL’s IACUC approved the challenge study, protocol 20-107.

## Author contributions

YS and JAB designed and interpreted the project. YS and JL processed samples and ran cellular assays. RZ, SKP, and YS performed Ab assays. LP and HA performed PRNT assays. JT and YS prepared the PLGA nanoparticle and other vaccines. INM, KWB, MM, and BMN performed pathology. HA, AC, RB, ET, JV, JG, TPT, TAC, MWB, and JK led the animal studies. YS, JAB, HA, LL, MGL, and LXW participated in study design and interpreted the experiments. DV and YS performed statistical analyses. YS and JAB wrote the manuscript with input from all the coauthors.

## Supplementary Material

Supplemental data

## Version 1 04/28/2021

In-Press Preview

## Version 2 05/24/2021

Electronic publication
