# Supplementary material for: Protection against SARS-CoV-2 infection by a mucosal vaccine in rhesus macaques
Source: JCI Insight. 2021 Apr 28;6(10):e148494. doi: 10.1172/jci.insight.148494 (PMC8262352; doi:10.1172/jci.insight.148494)
Supplement: Supplemental data [file jciinsight-6-148494-s132.pdf]

Supplementary data

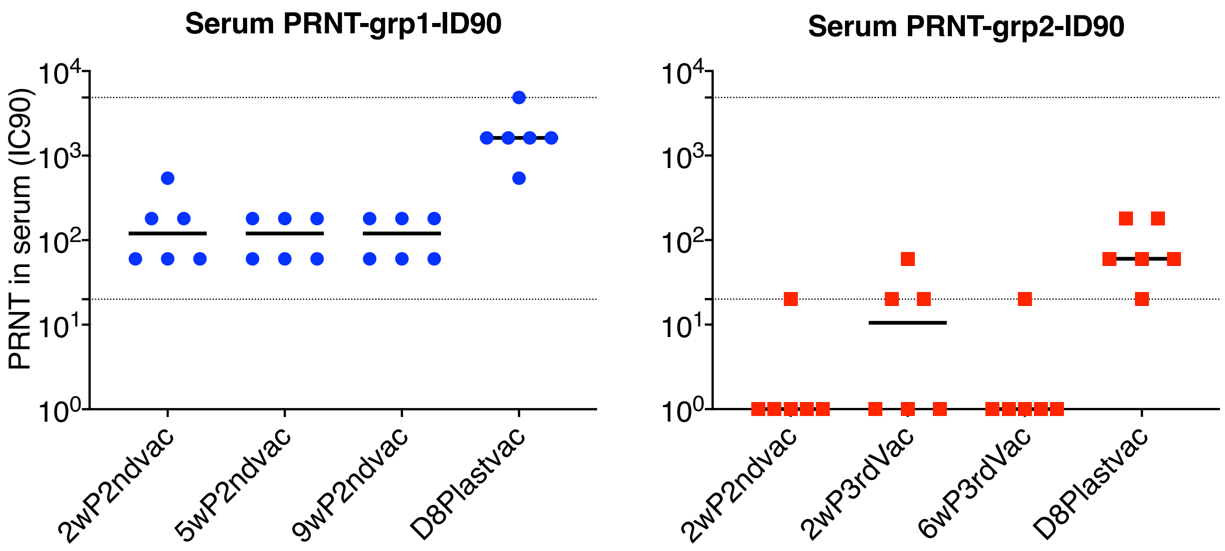

Supplementary Figure 1. The kinetics of PRNT neutralizing titers (ID90) in the vaccinated groups. Short lines and dashed lines show medians, the lower and upper assay limits respectively.

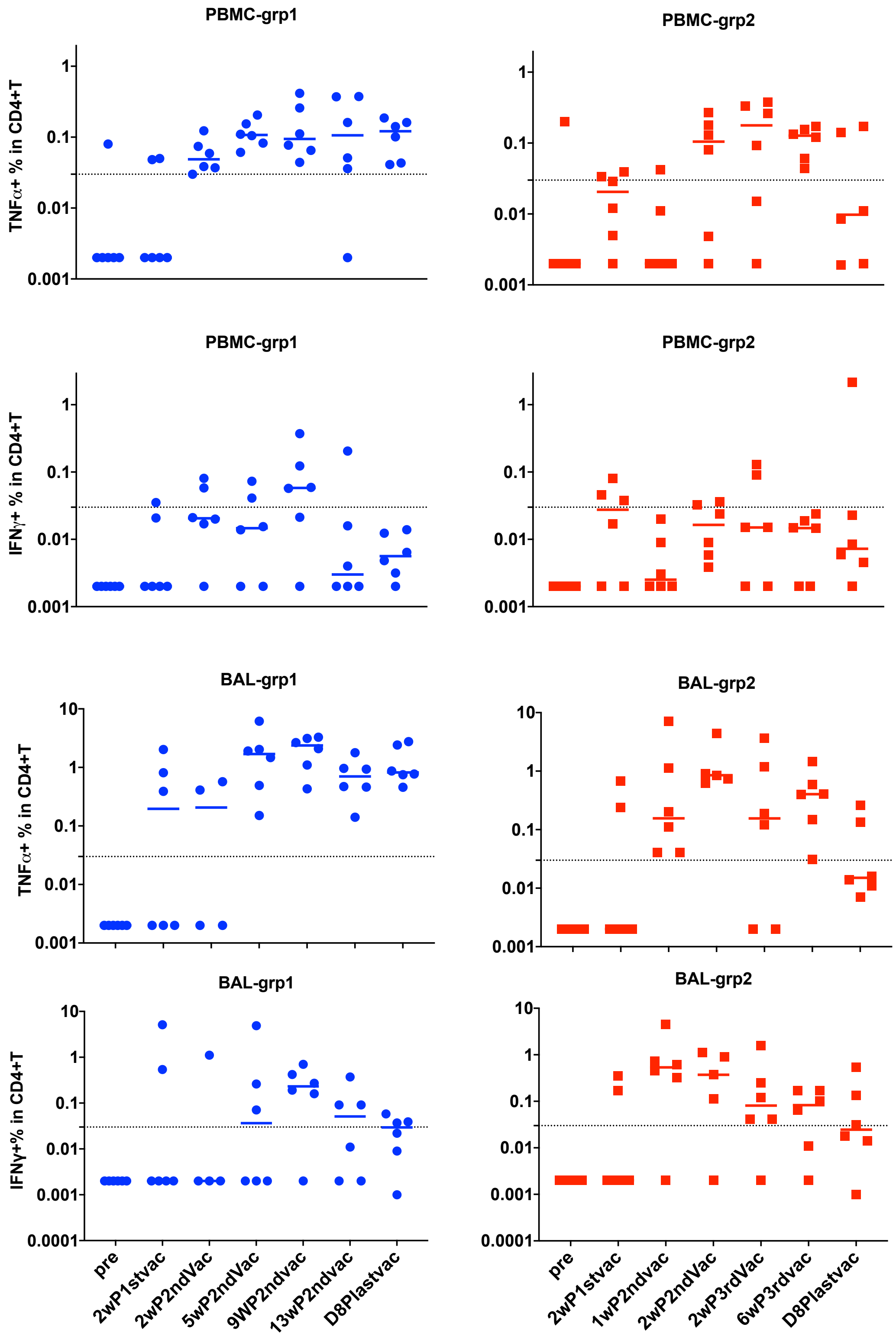

Supplementary Figure 2. Spike-specific TNFα+ or IFNγ+ CD4+ T cell responses in PBMC and BAL samples of the vaccinated animals. Short lines show medians.

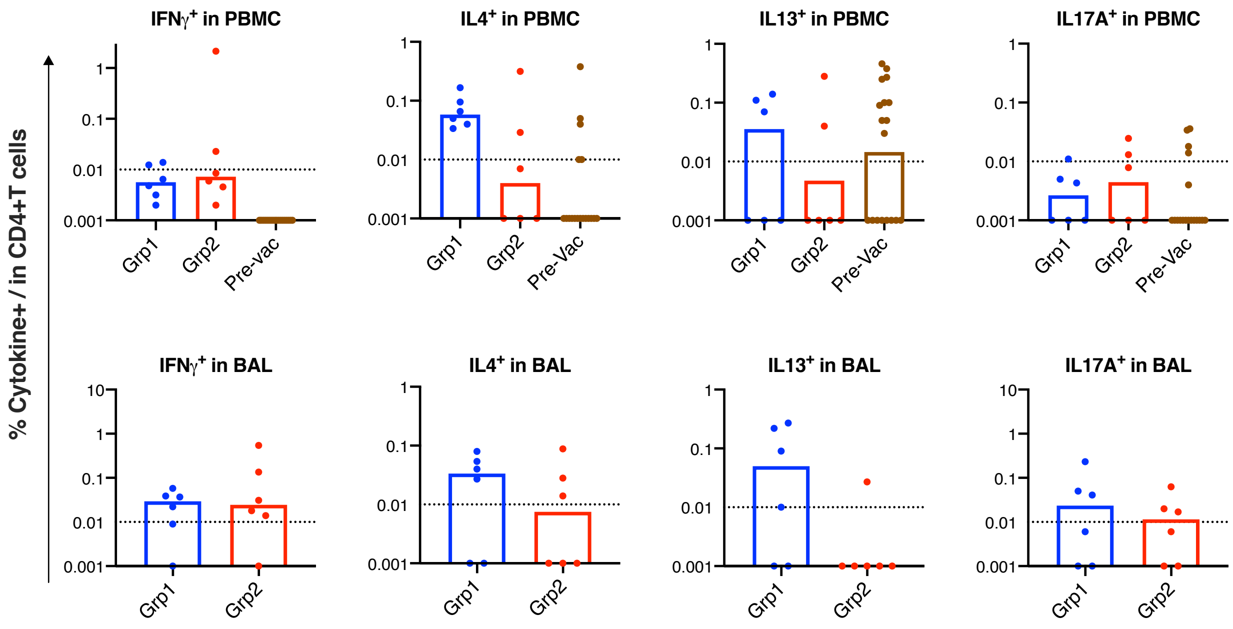

Supplementary Figure 3. Spike-specific Th subsets in PBMC and BAL samples of the vaccinated animals at Day8 post last vaccination. The dashed lines show the assay limit (bars are the medians). Medians are shown. Dashed lines are the threshold for positive responses.

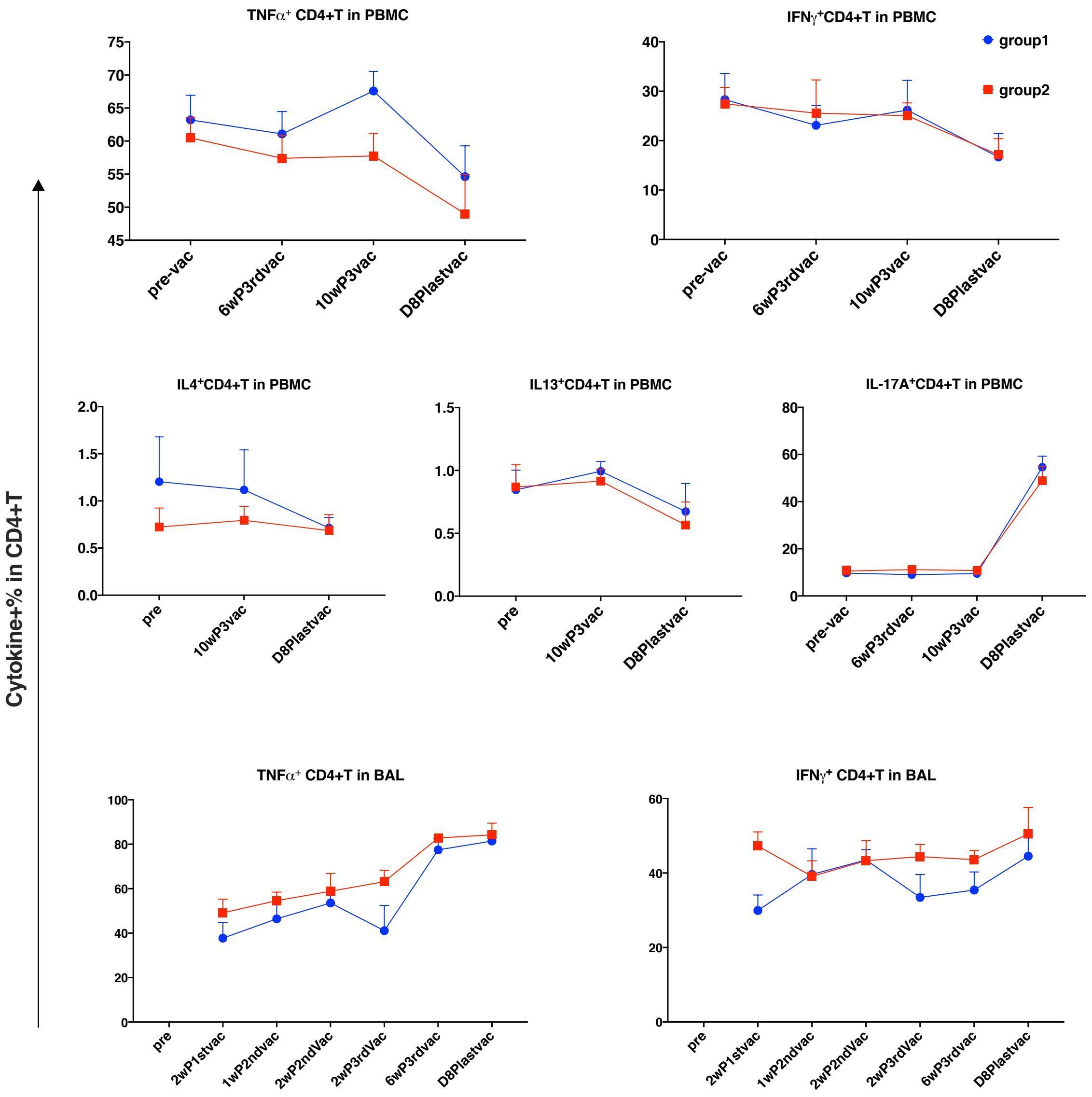

Supplementary Figure 4. The kinetics of PMA-stimulated Th1, Th2 and Th17 subsets in PBMCs and Th1 subsets in BALs of the vaccinated animals. Means with SEMs are shown.

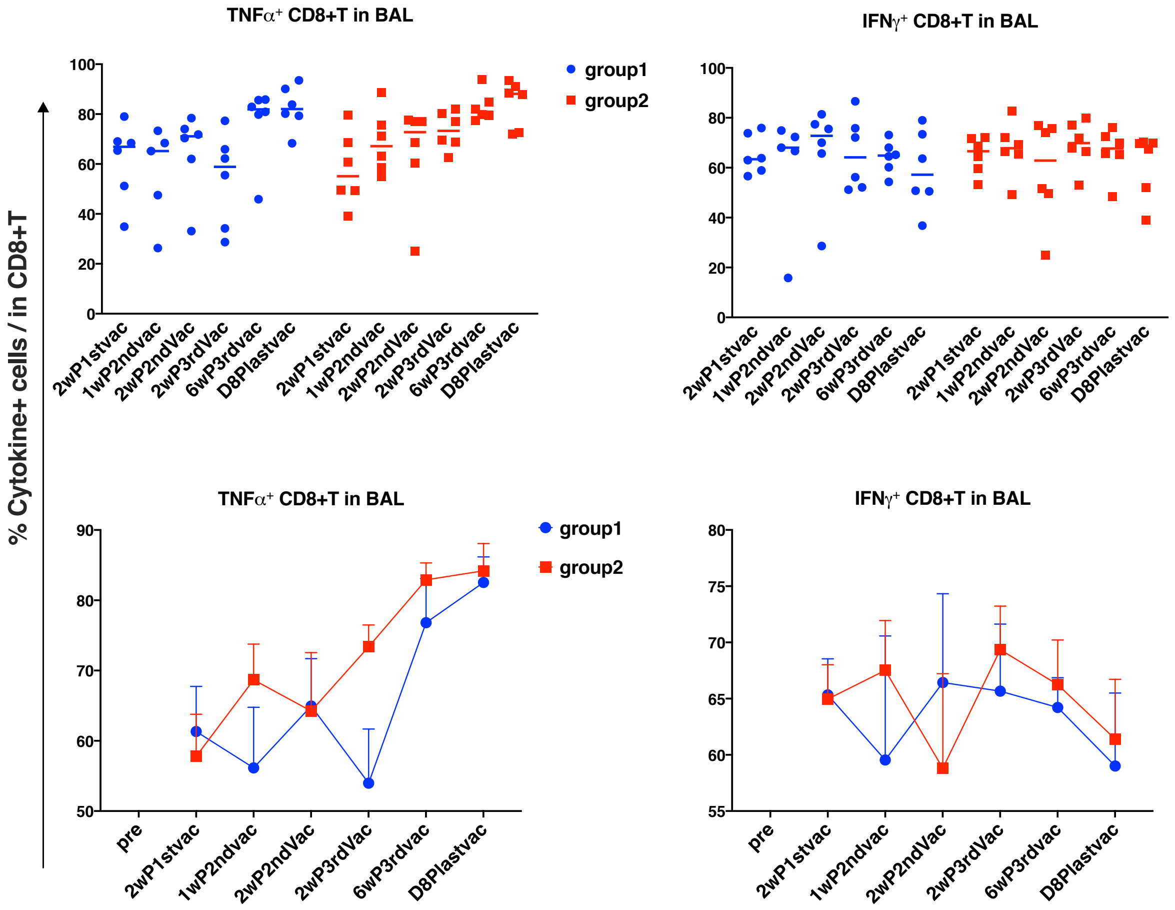

Supplementary Figure 5. The Tc1 immune responses stimulated by PMA in the BAL samples of the vaccinated animals. Medians are shown in the upper panels, means with SEMs are shown in the lower panels.

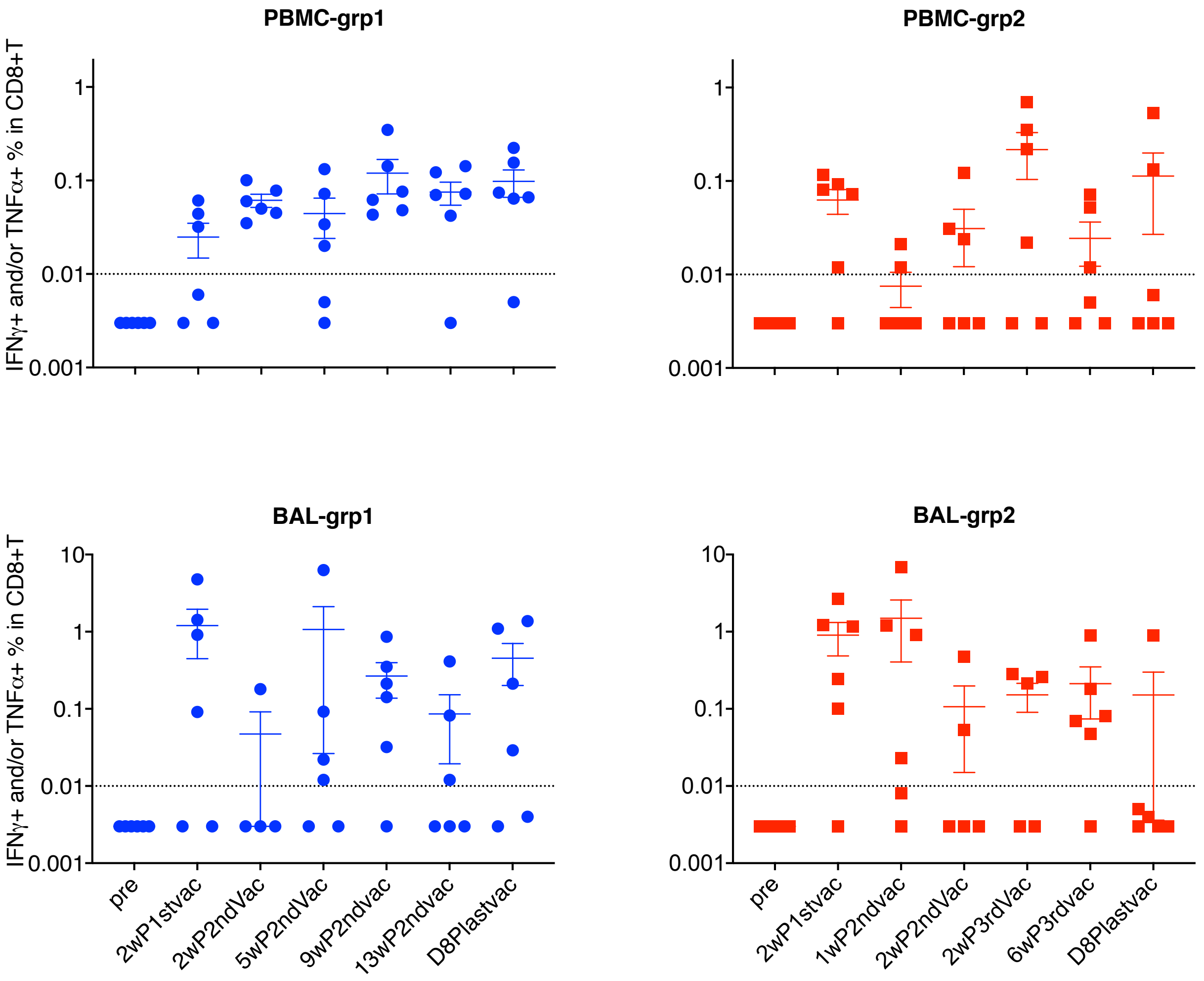

Supplementary Figure 6. Spike-specific TNFα+ and/or IFNγ+ CD8+ T cell responses in PBMC and BAL samples of the vaccinated animals. Medians are shown in the upper panels, means with SEMs are shown in the lower panels.

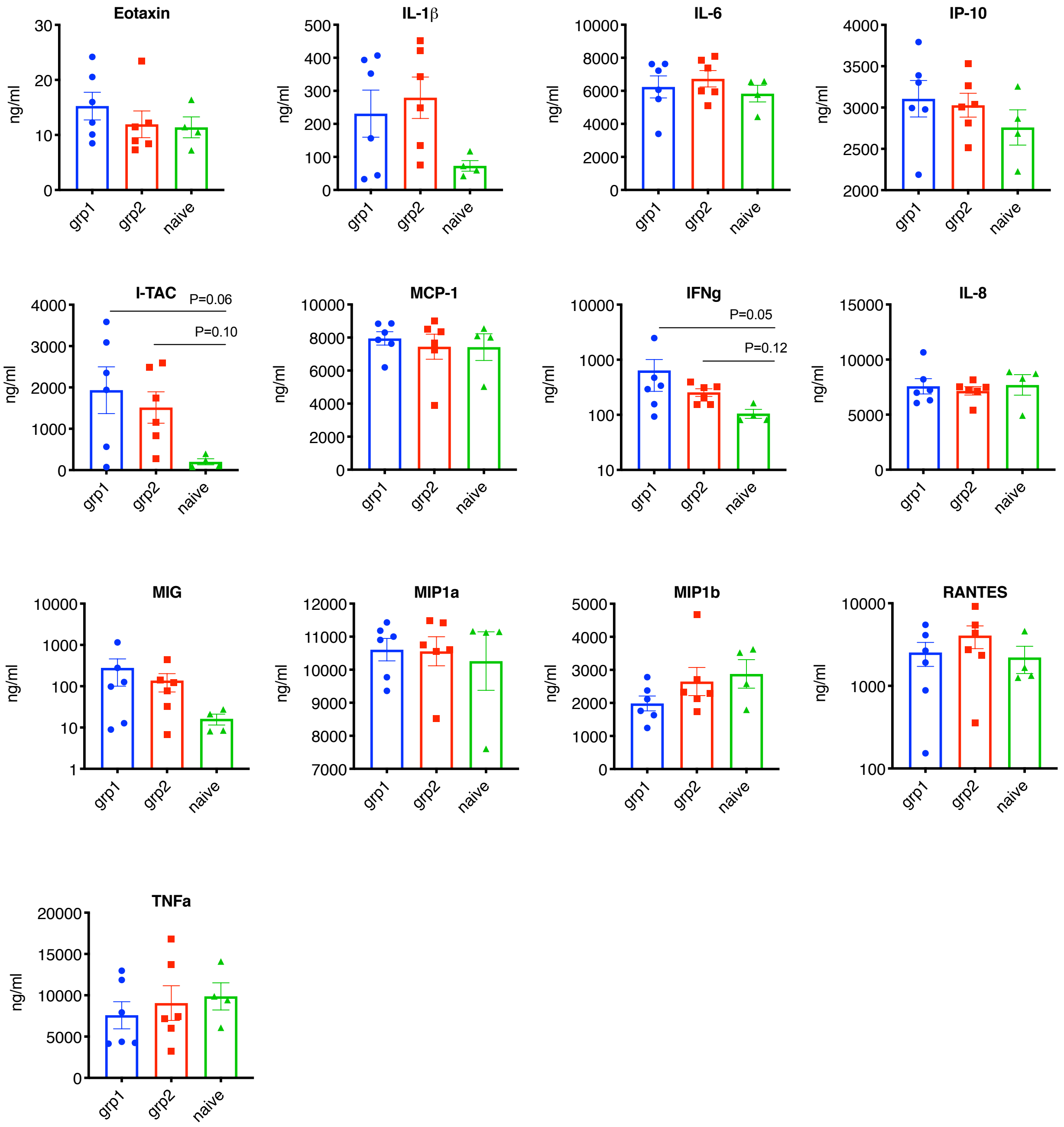

Supplementary Figure 7. Chemokines and cytokines production upon stimulation with polyI:C+S1 in the BAL samples from the vaccinated animals. BAL samples from 1-week post 2nd vaccination were treated with polyI:C+S1 protein for 18 hrs. before supernatant was collected for measurement of a total of 13 chemokines and cytokines. Means with SEMs are shown

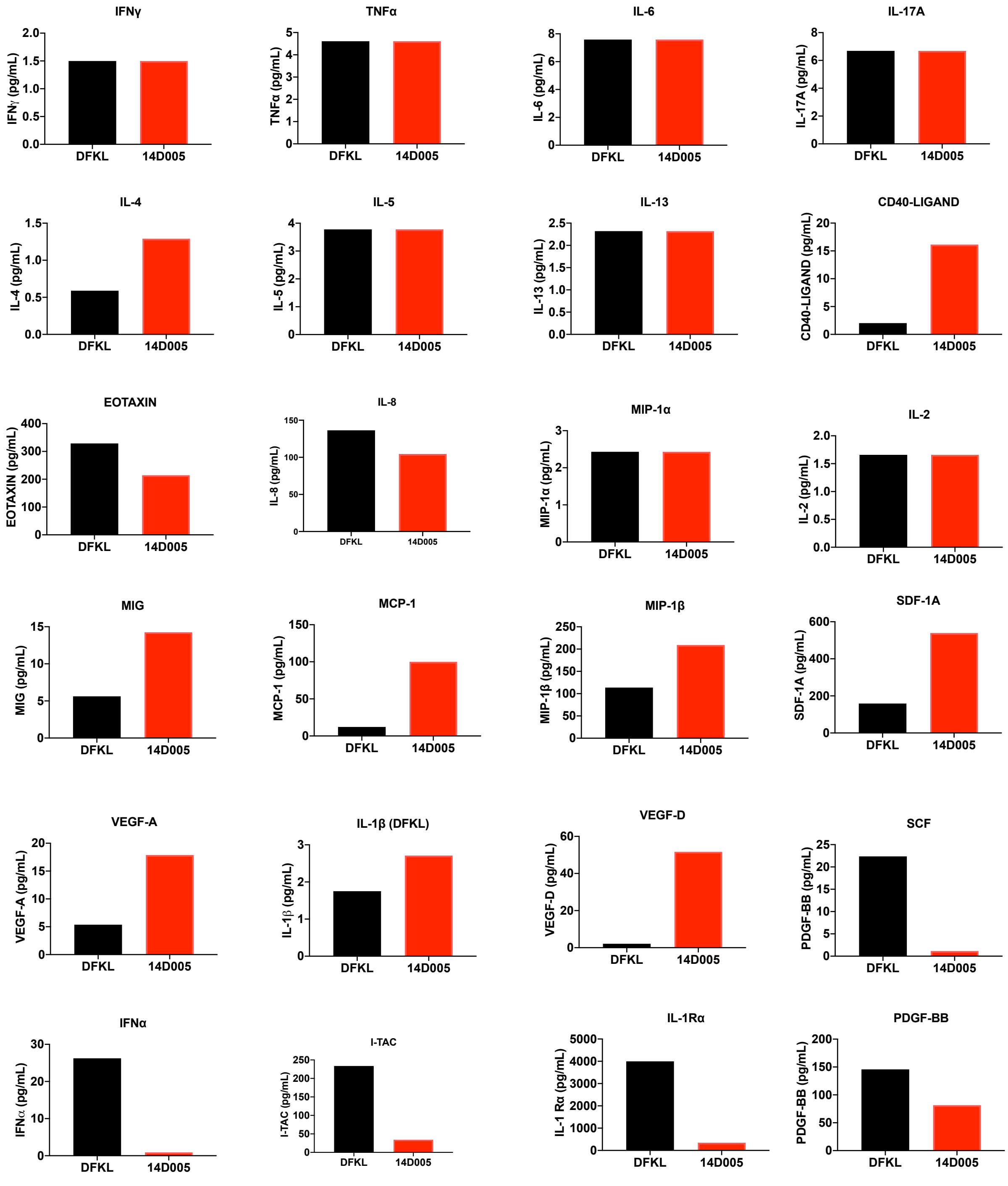

Supplementary Figure 8. Chemokines and cytokines production in the serum of two naïve control animal's two days after SARS-CoV-2 challenge. DFKL is a naïve control animal that never showed any viral load, while 14D005 showed viral load.

Supplementary Table1. Pathological summary of the animals challenged with SARS-Cov-2 virus

	Animal#	Age (years)	Necropsy Days post challenge	Lung H&E (Lc, Rmid, Rc)*	COVID IHC (Lc, Rmid, Rc)**	Comments#
Group1	DG9V	4	Day7	-;-;-	-;-;-	
	DGTK	4	Day7	-;-;+/-	-;-;-	Very mild, focal Alv hypercellularity
	DG9P	4	Day 7	-;-;-	-;-;-	
	DGA5	4	Day 10	-;-;-	-;-;-	
	DG8X	4	Day 10	-;+/-;-	-;-;-	
	DGLV	5	Day 10	-;+/-;+/-	-;-;-	
Group2	DGWN	4	Day7	-;-;-	-;-;-	
	DG1V	4	Day7	-;-;+/-	-;-;-	
	DG0Z	4	Day 7	-;-;-	-;-;-	
	DGD1	5	Day 10	-;-;-	-;-;-	
	DGXT	4	Day 10	+;+/-;+++	-;-;-	intraAlv; Type II hyper
	DGZC	4	Day 10	-;+/-;-	-;-;-	Small, mild, focal area of Type II hyper; otherwise minimal change
Naive	14D005	6	Day 7	+/-;+/-;+	-;-;+/-	Alv; intra Alv; Type II hyper; mild PV
	14D006	6	Day 7	+;+++;+	-;+/-;+/-	Changes Alv/ br; appeared more chronic with Type II hyper
	14D035	6	Day 7	+/-;-;+	-;-;-	Mild, focal Alv hypercellularity; mild PV inflammation
	14D043	6	Day 10	++;++;++	-;-;-	Changes Alv/br; intraAlv; appeared more chronic with very prominent Type II hyper
	14D041	6	Day 10	+/-;-;-	-;-;-	Changes were mild, focal, Alv hypercellularity
	DFKL	8	Day 10	+/-;+/-;+	-;-;-	Mild focal Alv hypercellularity; focal intraAlv infiltrates and mild, focal PV; mild, focal, (prominent) Type II hyper

*Inflammation score: <10% (tissue affected) = +/-; >10-<25% = +; >26-<50% = ++; >50%= +++
**COVID-19 viral Antigen (Ag) scoring: Minimal - absent = -; Minimally abundant (but clearly present)/ rare = +/-; Mildly abundant/ occasional = +; Moderately abundant =++; Abundant = +++

alveolar interstitium (Alv); intra-alveolar infiltrates (intraAlv); Bronchi (Br) or Bronchioles (br); Perivascular spaces (PV) or Type II pneumocyte hyperplasia (Type II). Left caudal (Lc); Right Middle (Rmid), and Right caudal (Rc) lobes.

Supplementary Table 2. Monoclonal antibodies used in this study.

Antibody	conjugated	clone	company
CD3	PE-cy7	SP34-2	BD
CD4	BV605	L200	BD
CD8	APC-cy7	RPA-T8	BD
CD28	FITC	CD28.2	Biolegend
CD95	PE-cy5	DX2	Biolegend
TNFα	Brilliant Violet 711™	MAb11	Biolegend
IFNγ	PE or PerCP	4S.B3	Biolegend
IL-4	Alexa Fluor® 647	8D4-8	Biolegend
IL-13	PE	JES 10-5A2.2	Miltenyi Biotech
IL-2	Brilliant Violet 785™	MQ1-17H12	Biolegend
IL-17A	Brilliant Violet 421™	BL168	Biolegend
CD14	Brilliant Violet 785™	M5E2	Biolegend
CD16	Brilliant Violet 421™	3G8	Biolegend
CD45	Alexa Fluor® 700	D058-1283	BD
